# Reviewer acknowledgement 2015

**DOI:** 10.1186/s40246-015-0026-2

**Published:** 2015-03-27

**Authors:** Vasilis Vasiliou

**Affiliations:** Yale School of Public Health, New Haven, CT USA

## Abstract

The Editors of *Human Genomics* would like to thank all our reviewers who have contributed to the journal in volume 8 (2014).

**Sükriye Ayter**

Turkey

**Emidio Capriotti**

United States of America

**Thomas Chen**

United States of America

**Shantanu Chowdhury**

India

**Michael Clark**

United Kingdom

**Carolien de Kovel**

Netherlands

**George Dedoussis**

Greece

**Alonso Fernandez-Guasti**

Mexico

**Paraskevi Goggolidou**

United Kingdom

**Leslie Jacqueline Greenberg**

South Africa

**Lin Hou**

United States of America

**Pei-Ing Hwang**

Taiwan

**Brian Jackson**

United States of America

**Dorothea Kasteleijn**

Netherlands

**Theodora Katsila**

Greece

**Rick Kittles**

United States of America

**Bernd Klaus**

Germany

**Chee Seng Ku**

Singapore

**Poh-San Lai**

Singapore

**Jun Li**

United States of America

**Christoph Lohmann**

Germany

**Frank Lovicu**

Australia

**Brian Meyer**

Saudi Arabia

**Jose Luis Millan**

United States of America

**Jonathan Mudge**

United Kingdom

**Daniel Nebert**

United States of America

**Michael Nothnagel**

Germany

**Giuseppe Novelli**

Italy

**Eric Pasmant**

France

**George Patrinos**

Greece

**Andrew Patterson**

United States of America

**Edward Perkins**

United States of America

**Mark Petrash**

United States of America

**Richard Radcliffe**

United States of America

**Michele Ramsay**

South Africa

**Peter Robinson**

Germany

**Michael Lee Robinson**

United States of America

**Elena Semina**

United States of America

**Jadranka Sertic**

Croatia

**Andrew Sharp**

Switzerland

**Clay Smith**

United States of America

**Poh Seng Tan**

Singapore

**Panagiotis Tsonis**

United States of America

**Carsten Wiuf**

Denmark

**Mathew Wright**

United Kingdom

**Shuhua Xu**

China

**Verena Zuber**

Norway

